# Editorial: Intersubjectivity: recent advances in theory, research, and practice

**DOI:** 10.3389/fpsyg.2023.1220161

**Published:** 2023-06-12

**Authors:** Theano Kokkinaki, Jonathan Delafield-Butt, Emese Nagy, Colwyn Trevarthen

**Affiliations:** ^1^Child Development and Education Unit, Department of Psychology, School of Social Sciences, University of Crete, Rethymno, Greece; ^2^Laboratory for Innovation in Autism and School for Education, University of Strathclyde, Glasgow, United Kingdom; ^3^School of Psychology, The University of Dundee, Dundee, United Kingdom; ^4^Department of Psychology, University of Edinburgh, Edinburgh, United Kingdom

**Keywords:** intersubjectivity, interbrain synchronization, embodied meaning-making, dyadic states of consciousness, loneliness, very premature birth, autism spectrum disorders, psychoanalytic developmental theory

Intersubjectivity describes the awareness of self and other's intentions and feelings in the dynamic sharing of minds acting in companionship, exchanging self-conscious intentions and emotional evaluations. Since 1960, when studies of infants disproved the theory of the young mind as a sensory-motor computer that is “conditioned” to learn facts symbolized in language, psychology now highlights the natural science of infant awareness, intelligence, intentions, and emotions and their sharing in embodied, non-verbal participation with others. This has fundamental implications for support of children's health, growth and learning in Psychology, Education, Pediatrics, Psychiatry and Psychotherapy and for mental health of infants, parents and teachers. It has strong confirmation by recent functional brain science. In this Research Topic entitled “*Intersubjectivity: recent advances in theory, research, and practice*,” we advance the science of intersubjectivity by bringing together new empirical studies, review, hypothesis and theory papers with advanced scholarship on the early emergence of human consciousness.

## Introduction

Intersubjectivity theory has had a long and fruitful career over the last three quarters of a century. Stemming from the anthropological work of Mead (1928) and Bateson (1975), the notion of intersubjectivity was adopted by infant and developmental psychologist to explain the pre-verbal dynamic sharing of interests, emotion, and intentions in face-to-face dyadic engagements between infant and parent (Trevarthen, 1998), and in developmental studies of and how children share in the rituals and spontaneous projects of home life that over the early years carries a child into the mastery of language (Stern, 2000; Nagy, 2008; Goodrich, 2010; Kokkinaki and Vitalaki, 2013; Trevarthen and Delafield-Butt, 2013, 2017; Delafield-Butt and Trevarthen, 2015).

Intersubjectivity theory is inherently an embodied science of mind (Trevarthen, 2012; Paolo and Jaegher, 2015), attending with scientific precision to the pre-verbal production of body movement to express and invent actions of thought in co-created meaning with another as the foundation of psychological developmental and later life productions of art, technology and tool use. Discovery of a culture in childhood involves learning a special form an embodied and enactive knowledge distinct from the learning theories of Pavlov and challenged by Bernstein (1967) who identified movement as the substrate of imaginative knowledge, and sharing ways of life. These rituals and their idiosyncratic experiences shared with caring and attentive others make up the stories that structure cultural learning (Bruner, 1977, 1990, 2004).

The science of intersubjectivity, with its understanding of feelings, embodiment, and companionship, is needed more today than it ever has been. With increasing attention to artificial intelligence and artificial worlds generated through the medium of technology, it is important to remind ourselves of the psychological and biological nature of how minds are shared, and come together make meaning and sense of the world in common purpose. They do this through sharing feelings, the affects and intentions that coordinate the body and compose the mind to work in step and in fluid efficiency with another mind. The peculiar psychobiology of intersubjectivity demonstrated long ago that this coming together of minds does so to generate something coherent and additional, more than the sum of its parts. This *inter-* (between or among, reciprocal) *subjectivity* (the experience of mind) is the foundation of human sharing, shared understanding, learning, and culture (Trevarthen and Hubley, 1978; Tronick, 2001).

That its study is credited in the early anthropology of Mead (1928) and Bateson (1971, 1975) is not to be missed. The science of intersubjectivity teaches us how minds are shared to generate micro (family) cultures and macro (societal) cultures, and all the nuances in between, especially in relation to health and learning, or pathology (Trevarthen et al., 2006). This science of intersubjectivity is only just beginning to reap rewards for societal and health benefit, and in the improvement of life for those with non-traditional and non-verbal voices and means of communication, such as infants or those with disruption to the typicality of fluid verbal speech.

In this special Research Topic, we have brought together a sample of important papers and perspectives that advance step-wise the science of intersubjectivity, from its underpinning neurobiology, its earliest development in premature birth, early childhood education and care, its cultural, psychological health, and human rights concerns. These papers contribute to improved understanding of our human nature, rich with affective resonances between us, and underpinning by a basic psychobiology that shares minds effortless between us. Knowing this with greater precision, and reflective conscious awareness enables better care and attention to those we seek to support.

Below we detail to give quick overview to the papers in this Research Topic.

## An interpersonal neurobiological model of the ontogeny of intersubjectivity

Schore (“*The interpersonal neurobiological of intersubjectivity*”) proposes an interpersonal neurobiological model of the ontogeny of intersubjectivity. He describes intersubjectivity as right-lateralized interbrain synchronization between the psychobiologically attuned mother and the developing infant while co-constructing engagement. These shared intersubjective interactions facilitate the maturation of the “social brain,” the temporoparietal junction of the right brain in the infant, leading to highly complex behavioral and interbrain synchronization. Based on recent brain laterality research, the clinical applications of this interpersonal neurobiological model of intersubjectivity are discussed.

## Precursors of effective and positive interactions as soon as birth in interactions of mothers with very preterm and full-term newborns

A pioneering study by Buil et al. explored how intersubjective communication can be facilitated by skin-to-skin positioning very preterm, 27-31 weeks-old gestational old newborns on their mothers, soon after birth (“*Skin-to-skin SDF positioning: the key to intersubjective intimacy between mothers and very preterm newborn—A pilot matched-pair case-control study*”). In a prospective, matched-pair study, Buil et al. placed the babies either in Supported Diagonal Flexion (SDF) positioning, where the pair can comfortably see each other, or in a Vertical position, where the mother sees mostly the top of her baby's head. The team then frame-by-frame coded the babies' states of consciousness, the babies' and mothers' vocalizations, and acoustic turn-taking, and found that in comparison to the vertical group, mothers in the SDF group offered a denser vocal envelope, with longer vocalizations, and the temporal proximity of the mothers' and babies' communication was greater. Overall, the authors propose that SDF promotes behavioral and brain-to-brain synchrony in premature baby-mother pairs.

Guellai and Streri (“*Mouth movements as possible cues of social interest at birth: new evidences for early communicative behaviors*”) showed that newborns as early as in the first days of life, produced more mouth movements when they saw a video of someone talking to them rather than a video of someone looking at them silently moving. Frequencies of mouth movements in the newborns were greater and the latency of the first mouth movement was shorter in front of a static face, compared to a dynamic face. The authors proposed that newborns already have a “sense of interaction” from birth, and they utilize motor feedback the interactive partner to time and shape their own responses.

## Dialogic book sharing as a privileged intersubjective space

Murray et al. focus on “Dialogic Book-Sharing” (DBS), an intersubjective form of using books with children. Through the evidenced-based justification on why involved processes (gaze following, pointing, naming and animating and special linguistic characteristics of book-sharing) are beneficial, the authors integrate evidence ranging from non-human primate communication to experimental and naturalistic studies of infant attention, cognitive processes and language. The authors argue that DBS is an intersubjective process of dynamic engagement and a natural propensity to share meaning. They highlight the importance of promoting DBS in effective training programmes, especially in disadvantaged populations as a powerful way to reduce economic inequality.

## Primary and secondary intersubjectivity: the continuity vs. discontinuity debate

Based on the fact that secondary intersubjectivity and the emergence of words are built on a foundation of primary intersubjectivity and with the aim to highlight the evolutionary origins of intersubjectivity, in their review paper, Terrace et al. (“*Intersubjectivity and the emergence of words*”) postulate that there is continuity between primary and secondary intersubjectivity and that both are necessary for the emergence of words. Even from birth, babies produce vocalizations called protophones that form part of the first vocal protoconversations, and are embedded in a multimodal, gestural turn-taking, that form a narrative, much before language develops. Taken the incremental changes measured in the development of most intersubjective abilities, Terrace et al. argue for a continuity, rather than a stage view of intersubjective development.

In Moll et al.'s hypothesis and theory paper, the authors contrasted two theses. The primary intersubjectivity thesis (PIT) highlights the humans' innate relational capacity from which social knowledge and understanding emerges. The shared intentionality thesis (SIT) postulates that human-unique forms of interaction develop through a cognitive revolution at 9-12 months of age when infants participate with others in acts of joint attention, imitative learning and cooperative action. The authors unified the strengths of the two theses and attempted to build a bridge between the PIT and the SIT by sketching how one expands into the other in a continuous process.

## Cross cultural aspects in resonance of timing, anticipation and empathy

In the cross-cultural study of aspects of intersubjectivity, Negayama et al. compared interactional synchrony in feeding between Japanese and Scottish mother-infant dyads. Different elements of timing, anticipation and empathetic mirroring underpinning mother-infant feeding process indicate two different cultural types of intersubjectivity in Japan and Scotland, which, according to the authors, become culturally and inter-generationally transmitted.

## Intersubjectivity as an antidote to stress

By using intersubjectivity as a hallmark of quality dyadic processes and within the framework of Free-Energy Principle, Ho et al. introduced a dyadic active inference model integrating basic dyadic concepts, to show the inverse relationship between stress and intersubjectivity. Using this model and through a theory-driven quantitative review, the authors supported their hypothesis that parenting intervention can effectively reduce parenting stress. Inspired by Buddhist Madhyamaka Philosophy, championed by Arya Nagarjuna and in order to elucidate the relation between intersubjectivity and wellbeing, the authors describe a relational worldview in terms of an abstract expression of Dependent Origination and applied this expression to the domains of physics, awareness, intersubjectivity and active inference.

## Interpreting loneliness within the theory of intersubjectivity

In her opinion paper, Galanaki (*Loneliness and intersubjectivity: A view from Trevarthen's theory*) argues that loneliness - the distress and social pain stemming from being alone - may be conceptualized within Trevarthen's developmental theory of intersubjectivity which focuses on innate-Other awareness.

According to the author, loneliness originates from humans' social brain, allocentric perception and innate dialogicity and thus may constitute a social or relational emotion. Social emotions are the causes of co-consciousness, of self-other awareness. Loneliness may be regarded as a moral emotion, as a motive for sympathy – a bridge between persons expressing their mutual assistance- belonging to our moral core. Moral emotions may facilitate sharing of meanings and purposes between sympathetic persons. A person's loneliness can be regarded as an innate intersubjective motive, a motive for seeking human company which floats in between persons and moves others. Solitude (an experience related to loneliness but distinct from it), evident even in very young infants, is a state of self-synchrony, a solitary state of reflection and contemplation. Early in life, private thinking and social communication co-exist in complementary ways.

Based on the implications of interpreting loneliness from the intersubjectivity view (existential disruptions, distortions of co-regulation and sharing, failure to co-construct meaning, to find cultural membership and to co-create a narrative about cosmos), the author concludes that “loneliness arises in a community of minds and is moderated by cultural membership and cultural sharing.”

## Intersubjectivity and rhythmic relating in atypical development

Heimann and Holmer (“*Neonatal imitation, intersubjectivity and children with atypical development: do observations on autism and down syndrome change our understanding?*”) showed that neonatal imitation was observed both in a case of a newborn who later developed ASD and in five, 1-month-old babies with DS. The authors suggest an updated model outlining two possible trajectories for children later receiving an ASD diagnosis. The authors conclude that imitation might not represent a useful predictor of a developmental deficit. Both children with ASD and DS are born with the ability for primary intersubjectivity, and the imitative ability documented in this research can enable them to enter into the first dialogues.

Based on the parameters of Communicative Musicality (pulse, quality and narrative), and on a thorough review of disruptions to social timing, sensorimotor timing and integration in autism, Daniel et al. (“*Rhythmic relating: bidirectional support for social timing in autism therapies*”) proposed Rhythmic Relating. Rhythmic Relating is a system and a skill set which aims to augment bidirectional communication and facilitate the predictive flow and just-a-head in time planning needed for social timing in child-centered therapeutic approach to interaction with individuals with autism.

Harrison and Tronick (“*Intersubjectivity: conceptual considerations in meaning-making with a clinical illustration*”) integrate three interrelated elements of meaning-making, that is meaning-making in interactions, making meaning with the body as well as the mind, and meaning-making within an open dynamic system, into a conceptual construct. The authors discuss the usefulness of this construct in psychoanalysis and provide clinical insights through the application of it as a dynamic evolving, multifaceted and non-linear “messy” process in the treatment of a 3-year-old child on the autistic spectrum.

## Intersubjectivity and pre-verbal children's rights

Våpenstad and Bakkenget (“*Pre-verbal children's participation in a new key. how intersubjectivity can contribute to understanding and implementation of child rights in early childhood*”) challenge the exclusion of preverbal infants' right to participate in relation to the United Nations Convention on the Rights of the Child (UNCRC) request for a definite way of involving infants. Based on the well-established ability of pre-linguistic children to communicate their interests, feelings and intentions through sympathetic rhythms, the authors propose that the voice of the infant can surface in adults verbal and musical narratives.

As the final article of the “*Intersubjectivity: recent Advances in theory, research, and practice” Research Topic* highlighted we all promote the recommendation of the United Nations on the Rights of the Child (2005) that research on methods for infant participation should be given the highest priority. Even the youngest children have the ability to participate, and must have the rights to be heard, accepted, respected. Colwyn Trevarthen, a leading pioneer in the field of child development and intersubjectivity warned that a failure to provide care and companionship to babies and toddlers inevitably results in major global issues in mental and physical wellbeing, that has further, immeasurable impact on economy, to form the foundation of our cultural values, how we respect and work with the infant to support his or her learning, growth and psychological development (Trevarthen et al., 2018a,b).

## Author contributions

All authors listed have made a substantial, direct, and intellectual contribution to the work and approved it for publication.

## References

[B1] Bateson M. C. (1971). The interpersonal context of infant vocalization. Quart. Prog. Rep. Res. Lab. Elect. 100, 170–176.

[B2] Bateson M. C. (1975). Mother-infant exchanges: the epigenesis of conversational interaction. Ann. New York Acad. Sci. 263, 101–113. 10.1111/j.1749-6632.1975.tb41575.x 1060428

[B3] Bernstein N. A. (1967). The Co-ordination and Regulation of Movements. Oxford: Pergamon Press.

[B4] Bruner J. S. (1977). “Early social interaction and language acquisition,” in Studies in Mother-Infant Interaction, ed H. R. Schaffer (New York, NY: Academic Press).

[B5] Bruner J. S. (2004). Life as narrative. Soc. Res. 71, 691–710. 10.1353/sor.2004.0045

[B6] Bruner J. S. (1990). Acts of Meaning. Cambridge: Harvard University Press.

[B7] Delafield-Butt J. T. Trevarthen C. (2015). The Ontogenesis of Narrative: From moving to meaning [Hypothesis and Theory]. Front. Psychol. 6, 01157. 10.3389/fpsyg.2015.01157 26388789 PMC4557105

[B8] Goodrich B. G. (2010). We do, therefore we think: time, motility, and consciousness. Rev. Neurosci. 21, 331–361. 10.1515/REVNEURO.2010.21.5.331 21280454

[B9] Kokkinaki T. Vitalaki E. (2013). Comparing spontaneous imitation in grandmother-infant and mother-infant interaction: a three generation familial study. Int. J. Aging Human Develop. 77, 77–105. 10.2190/AG.77.2.a 24416964

[B10] Mead M. (1928). Coming of age in Samoa; a psychological study of primitive youth for Western civilization. New York: W. Morrow and Company.

[B11] Nagy E. (2008). Innate Intersubjectivity: newborns' sensitivity to communication disturbance. Develop. Psychol. 44, 1779–1784. 10.1037/a0012665 18999338

[B12] Paolo E. D. Jaegher H. D. (Eds.). (2015). Towards an embodied science of intersubjectivity: Widening the scope of social understanding research. Front.Media. 3, 9. 10.3389./978-2-88919-529-9 PMC4345764 25784893

[B13] Stern D. N. (2000). The Interpersonal World of the Infant: A View from Psychoanalysis and Development Psychology (Second ed.). Oxford: Basic Books.

[B14] Trevarthen C. (1998). The concept and foundations of intersubjectivity. In S. Braten (ed.), Intersubjective Communication and Emotion in Early Ontogeny (pp. 15-46). Cambridge University Press.

[B15] Trevarthen C. (2012). Embodied human intersubjectivity: imaginative agency, to share meaning. J. Cogn. Semiot. IV, 6–56. 10.1515/cogsem.2012.4.1.626388789

[B16] Trevarthen C. Aitken K. J. Vandekerckhove M. Delafield-Butt J. Nagy E. (2006). “Collaborative regulations of vitality in early childhood: stress in intimate relationships and postnatal psychopathology,” in Developmental Psychopathology (pp. 65-126). John Wiley and Sons, Inc. 10.1002/9780470939390.ch2

[B17] Trevarthen C. Delafield-Butt J. Dunlop A. W. (eds.). (2018a). The Child's Curriculum: Working With the Natural Values of Young Children. Oxford: Oxford University Press, USA. 10.1093/oso/9780198747109.001.0001

[B18] Trevarthen C. Delafield-Butt J. T. (2013). “Biology of shared meaning and language development: regulating the life of narratives,” in The Infant Mind: Origins of the Social Brain, eds M. Legerstee, D. Haley, and M. Bornstein (pp. 167-199). Guildford Press.

[B19] Trevarthen C. Delafield-Butt J. T. (2017). “Development of consciousness,” in Cambridge Encyclopedia of Child Development, eds B. Hopkins, E. Geangu, and S. Linkenauger. (Cambridge University Press), (pp. 821-835) 10.1017/9781316216491.131

[B20] Trevarthen C. Dunlop A. W. Delafield-Butt J. (2018b). “The spirit of the child inspires learning in the community: how can we balance this promise with the politics and practice of education?” in The Child's Curriculum, eds C. Trevarthen, J. Delafield-Butt, and A. W. Dunlop. (Oxford University Press). 10.1093/oso/9780198747109.003.0017

[B21] Trevarthen C. Hubley P. (1978). “Secondary intersubjectivity: confidence, confiding and acts of meaning in the first year,” in Action, Gesture and Symbol, ed A. Lock (Academic Press), (pp. 183-229).

[B22] Tronick E. Z. (2001). Emotional connections and dyadic consciousness in infant-mother and patient-therapist interactions—Commentary on paper by Frank M. Lachmann. Psychoanal. Dialog. 11, 187–194. 10.1080/10481881109348606

[B23] United Nations on the Rights of the Child. (2005). “Convention on the Rights of the Child”. General Comment (No.7):Implementing Child Rights in Early Childhood. Geneva (Fortieth Session, 12-30 September).

